# Red blood cell transfusion within the first 24 hours of admission is associated with increased mortality in the pediatric trauma population: a retrospective cohort study

**DOI:** 10.1186/1752-2897-2-9

**Published:** 2008-10-20

**Authors:** Taylor J Stone, Paul J Riesenman, Anthony G Charles

**Affiliations:** 1 Department of Surgery, Division of Trauma and Critical Care Surgery, University of North Carolina School of Medicine, Chapel Hill, North Carolina, USA

## Abstract

**Background:**

Allogeneic red blood cell transfusion is associated with increased morbidity and mortality in adult trauma patients. Although studies have suggested that the adoption of a more restrictive transfusion strategy may be safely applied to critically ill adult and all-cause critically ill pediatric patients, recent developments in our understanding of the negative consequences of red blood cell transfusion have focused almost entirely on adult populations, while the applicability of these findings to the pediatric population remains poorly defined. The object of this study was to evaluate the effect of red blood cell transfusion within the first 24 hours following admission on mortality in pediatric trauma patients treated at our institution.

**Results:**

Age, race, and mechanism of injury did not differ between transfused and non-transfused groups, although there were significantly more female patients in the transfusion group (51 vs. 37%; p < 0.01). Shock index (pulse/systolic blood pressure), injury severity score, and new injury severity score were all significantly higher in the transfused group (1.21 vs. 0.96, 26 vs. 10, and 33 vs. 13 respectively; all p ≤ 0.01). Patients who received a red blood cell transfusion experienced a higher mortality compared to the non-transfused group (29% vs. 3%; p < 0.001). When attempting to control for injury severity, goodness-of-fit analysis revealed a poor fit for the statistical model preventing reliable conclusions about the contribution of red blood cell transfusion as an independent predictor of mortality.

**Conclusion:**

Red blood cell transfusion within the first 24 hours following admission is associated with an increase in mortality in pediatric trauma patients. The potential contribution of red blood cell transfusion as an independent predictor of hospital mortality could not be assessed from our single-institution trauma registry. A review of state-wide or national trauma databases may be necessary to obtain adequate statistical confidence.

## Background

Nonoperative management of blunt traumatic injury is now a widely accepted practice in hemodynamically stable patients[1,2]. The transfusion of allogeneic packed red blood cells (PRBCs) is employed to attenuate reductions in hemoglobin. An increase in serum hemoglobin will increase the oxygen-carrying capacity of the blood, which theoretically provides more oxygen to vital tissues malperfused in the shock state. However, recent studies have associated adverse hospital outcomes with therapeutic blood transfusions in adult patients. Allogeneic blood transfusion has been reported to be an independent predictor of hospital mortality in adult trauma patients [1-4]. Additionally, PRBC transfusion is associated with an increased risk of infection [5], multisystem organ failure (MSOF),[6,7] and systemic inflammatory response syndrome (SIRS)[4] in adult trauma patients. Additional studies have suggested that the adoption of a more restrictive transfusion strategy may be safely applied to critically ill adult patients,[8,9] and all-cause critically ill pediatric patients[10].

Recent developments in our understanding of the negative consequences of PRBC transfusion have focused almost entirely on adult populations, while the applicability of these findings to the pediatric population remains poorly defined. The purpose of this study was to examine the effect of blood transfusion within the first 24 hours of admission on hospital mortality in the pediatric trauma patient population.

## Results

### Patients

Over the 8-year study period, 1639 pediatric trauma patients were admitted to our trauma center of which 106 (6.5%) received at least one PRBC transfusion within the first 24 hours of admission. Patient characteristics of the overall cohort, as well as transfused and non-transfused groups are presented in Table 1. Age, race, and mechanism of injury did not differ between groups, although there were significantly more female patients in the transfusion group.

**Table 1:** Patient characteristics of transfused and non-transfused groups

	**Total Cohort**	**Transfused**	**Non-Transfused**	**p**
**n**	1639	106	1533	
**Age (yrs)**	7.8 ± 5.0	7.4 ± 5.5	7.8 ± 4.9	0.331
**Gender**				
Males	1021 (62%)	52 (49%)	969 63%)	0.004
Females	618 (38%)	54 (51%)	564 (37%)	
**Race**				
White	950 (58%)	52 (49%)	898 (59%)	0.056
African-American	399 (24%)	31 (29%)	368 (24%)	0.224
Hispanic	185 (11%)	17 (16%)	168 (11%)	0.110
Other	105 (6%)	6 (6%)	99 (6%)	0.746
**Mechanism of Injury**				
Blunt	1520 (93%)	98 (92%)	1422 (93%)	0.906
Penetrating	118 (7%)	7 (7%)	111 (7%)	
Burn	1 (0%)	1 (1%)	0 (0%)	

### Blood Transfusion

Physiologic and anatomic measures of injury severity of transfused and non-transfused groups are presented in Table 2. Shock index (SI), injury severity score (ISS), and new injury severity score (NISS) were all significantly higher in the transfused group. Assessment of the number of PRBC units, or volume of individual units transfused could not be reliably quantified due to the large number of patients who presented as transfers from referring hospitals.

**Table 2:** Physiologic and anatomic measures of injury severity

	**Transfused (n = 106)**		**Non-Transfused (n = 1533)**		
	Mean ± SD	n (%)^1^	Mean ± SD	n(%)^1^	p
**Physiologic**					
Heart Rate	131 ± 35	92 (87%)	112 ± 29	1292 (84%)	< 0.001
Respiratory Rate	10 ± 14	83 (78%)	21 ± 11	1216 (79%)	< 0.001
SBP	115 ± 31	85 (80%)	123 ± 20	1178 (77%)	< 0.001
Shock Index	1.2100 ± 0.547	84 (79%)	0.961 ± 0.878	1168 (76%)	0.010
					
**Anatomic**					
AIS Abdomen	2.737 ± 0.921	38 (36%)	2.304 ± 0.739	217 (14%)	0.002
AIS Chest	3.413 ± 0.777	46 (43%)	3.038 ± 0.887	209 (19%)	0.009
AIS Extremities	2.618 ± 0.561	55 (52%)	2.498 ± 0.571	636 (41%)	0.136
AIS Face	1.913 ± 0.596	23 (22%)	1.690 ± 0.586	203 (13%)	0.085
AIS Head and Neck	4.231 ± 0.952	78 (73%)	3.162 ± 1.132	659 (43%)	< 0.001
AIS Skin	1.273 ± 0.550	22 (21%)	1.154 ± 0.376	377 (25%)	0.162
ISS	26 ± 13	106 (100%)	10 ± 9	1530 (99.8%)	< 0.001
NISS	33 ± 18	106 (100%)	13 ± 13	1530 (99.8%)	< 0.001

AIS: abbreviated injury scale; ISS: injury severity score; NISS: new injury severity score; ^1^number and % reporting for the respective data point.

### Hospital Outcomes

Patients who received a PRBC transfusion experienced a longer mean intensive care unit (ICU) length of stay and were significantly more likely to stay in the ICU > 1 day. (Table 3) Additionally, patients in the PRBC transfusion group also experienced a longer mean overall hospital length of stay and a higher hospital mortality (29.2% vs. 2.7%, p < 0.001). The crude mortality odds ratio (OR) for the effect of PRBC transfusion on mortality was 14.67. When accounting for observations censored due to missing SI and/or ISS values (389 patients), the mortality odds ratio was 16.61. However, by censoring patients with missing values, we decreased our transfused and non-transfused patient populations by approximately 20% and 23% (n = 85 and n = 1178) respectively. Logistic regression analysis on the remaining cohort revealed that age, gender, and race did not have a significant effect on mortality when accounting for PRBC transfusion (p = 0.461, 0.403, 0.642 and OR estimates = 0.982, 1.247, 1.059 respectively). Although PRBC transfused patients had a higher crude mortality OR, goodness-of-fit analysis revealed dissimilar Deviance and Pearson values for models incorporating SI and/or ISS scores, indicating a poor fit for these statistical models. The cohort failed to provide physiologic and anatomic injury data which could be modeled reliably to allow conclusions about the contribution of PRBC transfusion as an independent predictor of mortality.

**Table 3:** Hospital Outcomes

	**Transfused (n = 106)**	**Non-Transfused (n = 1533)**	**p**
ICU Length of Stay (days)^1^	6.1 ± 6.7	1.1 ± 3.8	< 0.001
ICU Length of Stay ≥ 1 Day^2^	96 (91%)	458 (30%)	< 0.001
Hospital Length of Stay (days)^1^	12.9 ± 12.3	4.5 ± 7.3	< 0.001
Mortality^2^	31 (29.2%)	42 (2.7%)	< 0.001

^1^mean ± SD; ^2 ^number and % reporting for the respective data point

## Discussion

Following trauma, cause of death varies according to the time after the initial insult. In a study looking at the etiologies of traumatic deaths, Sauaia et al,[11] found that 34% of deaths occurred in the pre-hospital setting and 66% occurred in the hospital. Of the patients admitted to the hospital, 81% died within the first 48 hours (acute), 6% within 3–7 days (early), and 14% after seven days (late). While central nervous system injury and exsanguination were the most frequent mechanisms responsible for acute and early deaths, MSOF was the most common cause of late death (61%).

In the acute setting PRBC transfusion can be lifesaving; however, consequences of transfusion may paradoxically result in an increased loss of life from organ failure. PRBC transfusion is often used to attenuate hemoglobin reduction from injury-associated blood loss, but evidence has been accumulating that suggests PRBC transfusion is an independent predictor of post-injury infection[5] and MSOF[6,7], as well as mortality, in both adult trauma [1-4,12] and pediatric critically ill patients[12]. In adult ICU patients, a multiple-center protocol that limited blood transfusion demonstrated reduced in-hospital mortality rate, cardiac complication rate, and organ dysfunction compared to a liberal transfusion group, lending evidence that limiting blood transfusion may eliminate some deaths from organ failure[9]. Although a recent study by Lacroix et al. suggests that a restrictive transfusion strategy can safely be employed a pediatric ICU population, no reductions in MSOF or other secondary outcomes were demonstrated over a more liberal transfusion strategy[10].

Although the hospital mortality rate was significantly higher in our transfused group, patients within this group were more severely injured as measured by physiologic (SI) and anatomic (ISS) measures of injury severity. Over the 8-year study period, only 106 pediatric trauma patients who received a PRBC transfusion within the first 24 hours of admission were identified. Additionally, the removal of patients missing SI or ISS values further reduced the number of observation in this group by approximately 20% (n = 85 patients). All patients having SI and ISS values were included in the final analysis regardless of injury score in order to try to evaluate the effect of transfusion on the entire pediatric trauma population, because we were also interested in outcomes involving lesser injured patients (as determined by the SI and ISS values). However, the small sample size in the transfused group statistically limited our ability to evaluate the independent effects of PRBC transfusion on mortality, as goodness-of-fit analysis on statistical models incorporating SI and/or ISS demonstrated that these models were unreliable. Therefore, a review of state-wide or national trauma databases may be necessary to obtain adequate statistical confidence to determine the independent effects of PRBC on mortality in the pediatric trauma patient population.

Our study suffered from a number of limitations. As a tertiary referral center, many of the patients in this study arrived as transfers from referring institutions. Incomplete transfer records limited our ability to quantify the volume of blood transfused, as well as determine the age of the blood products received. Furthermore the study period also bridged our transition over to exclusively using leukocyte-depleted packed red cells. Age of transfused blood correlates with a higher risk of MOF in trauma patients[7] and also increases mortality in patients with severe sepsis[13]. Stored blood undergoes mechanical changes and older blood may obstruct capillary blood flow, predisposing to tissue ischemia and infection[14]. A previous study at our institution demonstrated that in adult patients, the impact of blood transfusion on mortality is dose dependent, varying as a function of the number of units of PRBCs transfused[2]. These are important variables that have been appreciated in this field of study, which were unable to be addressed directly in this cohort of patients.

## Conclusion

Red blood cell transfusion in the first 24 hours following admission is associated with an increase in mortality in pediatric trauma patients. The potential contribution of red blood cell transfusion as an independent predictor of hospital mortality could not be assessed from our single-institution trauma registry. A review of state-wide or national trauma databases may be necessary to obtain adequate statistical confidence. Future research is needed to discover the mechanisms underlying poor transfusion outcomes in both the adult and pediatric populations.

## Methods

### Patients

The study was approved by the Institutional Review Board of the University of North Carolina. Over an eight-year period (1998–2006), trauma patient admissions were retrospectively reviewed from an established and verified trauma registry. All pediatric patients (≤ 16 years of age) sustaining blunt or penetrating trauma were included in the analysis and classified into transfused (at least one allogeneic PRBC transfusion within the first 24 hours after admission), and non-transfused groups.

Retrospectively acquired data from the trauma registry included age, gender, race, mechanism of injury (MOI), admission systolic blood pressure (SBP), heart rate (HR), and respiratory rate (RR). The shock index (SI) was calculated using the admission HR and SBP (SI = HR/SBP)[15] and represented admitting physiologic injury. Injury Severity Score (ISS), New Injury Severity Score (NISS), ICU length of stay, hospital length of stay, and hospital mortality were also obtained from registry data. Confirmation of patients identified as having received a blood transfusion within the first 24 hours was performed by reviewing individual patient records.

No specific transfusion protocol was present during the time of this study. Nonoperative management and PRBC transfusion were performed at the discretion of the attending trauma surgeon initially evaluating the patient or the attending intensivist once the patient was transferred to the ICU. In general, hemodynamic instability, failure to respond to resuscitation, and ongoing blood loss were common indications for transfusion.

### Statistical analysis

Univariate analysis was used to identify variables that were associated with blood transfusion, ICU length of stay, hospital length of stay, and hospital mortality. Chi-squared test was used to compare population proportions and two-tailed p values are reported. Student's *t *test was used for continuous variables. Data are expressed as mean ± standard deviation and comparison between groups is referred to as being statistically significant if p ≤ 0.05. Multivariate logistic regression was used to model the independent effect of blood transfusion on overall hospital mortality. Statistical methods and results were reviewed by an independent statistician.

## Competing interests

The authors declare that they have no competing interests.

## Authors' contributions

TS collected data, participated in the statistical analysis, and drafted the manuscript. PR collected data, participated in the design of the study, performed the statistical analysis, and helped draft the manuscript. AC conceived of the study and participated in its design and coordination, and helped to draft the manuscript. All authors read and approved the final manuscript.
